# Complete Genome Sequences of Six Measles Virus Strains

**DOI:** 10.1128/genomeA.00184-18

**Published:** 2018-03-29

**Authors:** My V. T. Phan, Claudia M. E. Schapendonk, Bas B. Oude Munnink, Marion P. G. Koopmans, Rik L. de Swart, Matthew Cotten

**Affiliations:** a Department of Viroscience, Erasmus MC, Rotterdam, The Netherlands

## Abstract

Genetic characterization of wild-type measles virus (MV) strains is a critical component of measles surveillance and molecular epidemiology. We have obtained complete genome sequences of six MV strains belonging to different genotypes, using random-primed next generation sequencing.

## GENOME ANNOUNCEMENT

Measles is one of the most contagious human diseases and is still responsible for considerable childhood morbidity and mortality. The causative agent, measles virus (MV), is an enveloped virus with a negative-sense single-stranded RNA genome and is a member of the genus Morbillivirus within the family Paramyxoviridae (1). MV genomes are relatively stable; the virus consists of a single serotype, and live-attenuated MV vaccines developed in the 1960s still confer protection against currently circulating wild-type MV strains. However, the MV genome also contains a number of variable regions that have been used to assign 8 genetic clades (A to H), which have been further subdivided into 24 genotypes (or subtypes) (2). The MV genome is typically 15,894 nucleotides (nt) in length, and it encodes 6 structural proteins (N, P, M, F, H, and L) and two nonstructural proteins (V and C).

Viral isolates of circulating wild-type MV strains provide an important resource for virology and vaccine development. In this work, we have obtained complete genome sequences for the following six wild-type MV strains: MVi/Khartoum.SUD/34.97/2 [B3], a strain endemically circulating in Khartoum (Sudan) in 1997 (3); MVi/Bilthoven.NLD/1991 [C2], a strain isolated during a measles outbreak in The Netherlands in 1991 (4); MVi/Amsterdam.NLD/19.11 [D4], an unpublished import case into The Netherlands isolated in 2011 from a patient who had traveled to Greece; MVi/Dodewaard.NLD/29.13 [D8], an isolate obtained during a large measles outbreak in The Netherlands in 2013 (5); MVi/Amsterdam.NLD/49.97 [G2], an isolate obtained from a secondary case in a hospital outbreak in 1997 (6); and MVi/Amsterdam.NLD/27.97, a virus isolated from a measles patient in 1997 with a recent history of travel to China (7). All viruses were isolated in Epstein-Barr virus-transformed human B-lymphoblastic cell lines, followed by short-term culture (maximum 3 passages) in Vero cells expressing human CD150 (8). All cultures were confirmed negative for Mycoplasma spp. All virus isolates are available via the European Virus Archive (https://www.european-virus-archive.com).

Total viral nucleic acid was extracted from 6 virus cultures (with titers between 10^5^ and 10^7^ 50% tissue culture infective dose [TCID_50_]/ml), using Bioke extraction reagents (Leiden, The Netherlands). Extracted RNA was reverse transcribed and second-strand synthesis was performed using random primers as previously described (9), followed by sequencing on the Ion Torrent S5XL platform to generate 2.2 × 10^6^ to 2.8 × 10^6^ 400-nt reads per sample. Raw reads were trimmed from the 3′ end to a median Phred score of 30 and minimum length of 75 nt using the Quality Assessment of Short Read (QUASR) package (10), and then *de novo* assembled using SPAdes version 3.11 (11).

Six complete MV genomes were obtained, including the first full genome for MV genotype C2. Of note, one MV genome (MVi/Amsterdam.NLD/19.11 [D4]) had a 6-nucleotide insertion, giving rise to a full genome length of 15,900 nt and complying with the rule of six for replication competency in MV (12). These sequence data will provide a useful reference for measles surveillance and for studies to better understand MV evolution and biology.

### Accession number(s).

The MV sequences described in this study have been deposited in GenBank under the accession numbers MG912589 to MG912594.
